# Type I Interferons in Systemic Autoimmune Diseases: Distinguishing Between Afferent and Efferent Functions for Precision Medicine and Individualized Treatment

**DOI:** 10.3389/fphar.2021.633821

**Published:** 2021-04-14

**Authors:** François Chasset, Jean-Michel Dayer, Carlo Chizzolini

**Affiliations:** ^1^ Department of Dermatology and Allergology, Faculty of Medicine, AP-HP, Tenon Hospital, Sorbonne University, Paris, France; ^2^ Emeritus Professor of Medicine, School of Medicine, Geneva University, Geneva, Switzerland; ^3^ Department of Pathology and Immunology, School of Medicine, Geneva University, Geneva, Switzerland

**Keywords:** interferon, systemic lupus erythematosus (SLE), genetic polymorphism, interferon-stimulated genes (ISGs), polymorphonuclear neutrophils (PMN), keratinocytes, autoantibody (autoAb), systemic autoimmune diseases (SADs)

## Abstract

A sustained increase in type I interferon (IFN-I) may accompany clinical manifestations and disease activity in systemic autoimmune diseases (SADs). Despite the very frequent presence of IFN-I in SADs, clinical manifestations are extremely varied between and within SADs. The present short review will address the following key questions associated with high IFN-I in SADs in the perspective of precision medicine. 1) What are the mechanisms leading to high IFN-I? 2) What are the predisposing conditions favoring high IFN-I production? 3) What is the role of IFN-I in the development of distinct clinical manifestations within SADs? 4) Would therapeutic strategies targeting IFN-I be helpful in controlling or even preventing SADs? In answering these questions, we will underlie areas of incertitude and the intertwined role of autoantibodies, immune complexes, and neutrophils.

## Introduction

**FIGURE 1:**
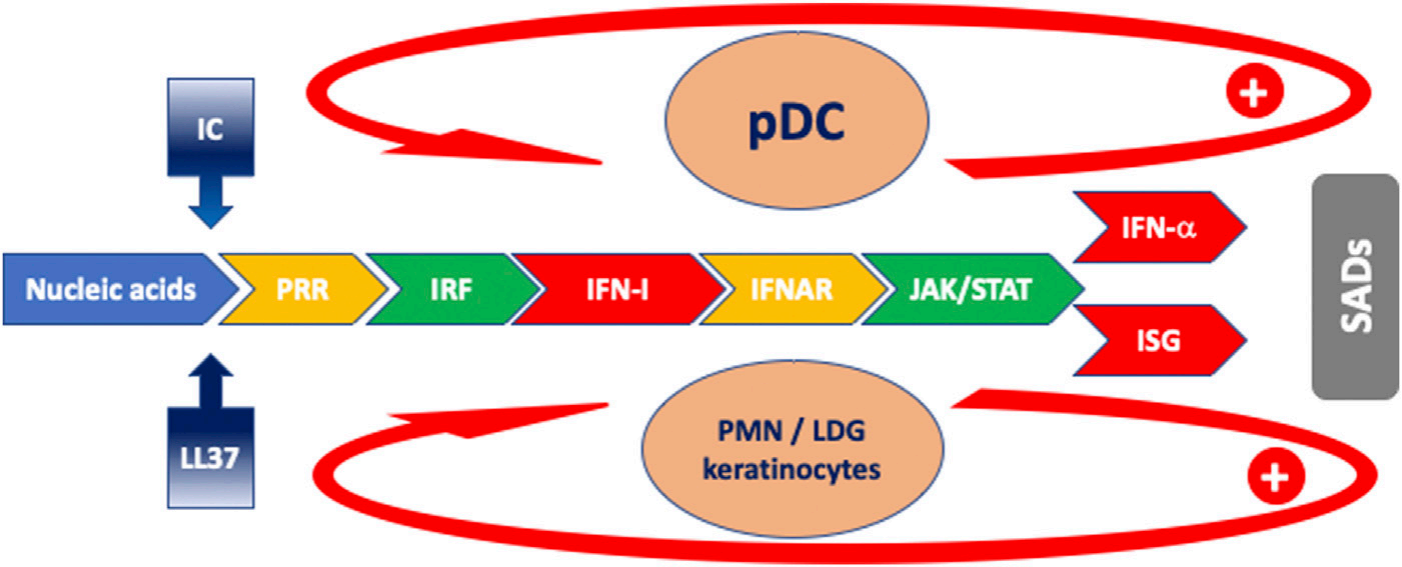
Schematic model of the cascade of events characterizing the IFN response in SADs. Blue: antigen drive; gradient blue: facilitator mechanisms for antigen uptake; yellow: receptors; green: intracellular signaling; red: IFN and ISG. The arrows indicate feed-forward regulatory mechanisms. Orange ovals: main cells implicated in IFN-I production. Abbreviations: IC: immune complexes; IFNAR: interferon-alpha/beta receptor; ISG: interferon-stimulated gene; ISRE: IRF: interferon regulatory factor; JAK: Janus kinase; LDG: low-density granulocyte; LL-37: cathelicidin-37; pDC: plasmacytoid dendritic cell; PMN: polymorphonuclear neutrophil; PRR: pattern recognition receptor; SADs: systemic autoimmune diseases; STAT: signal transducer and activator of transcription.

**FIGURE 2:**
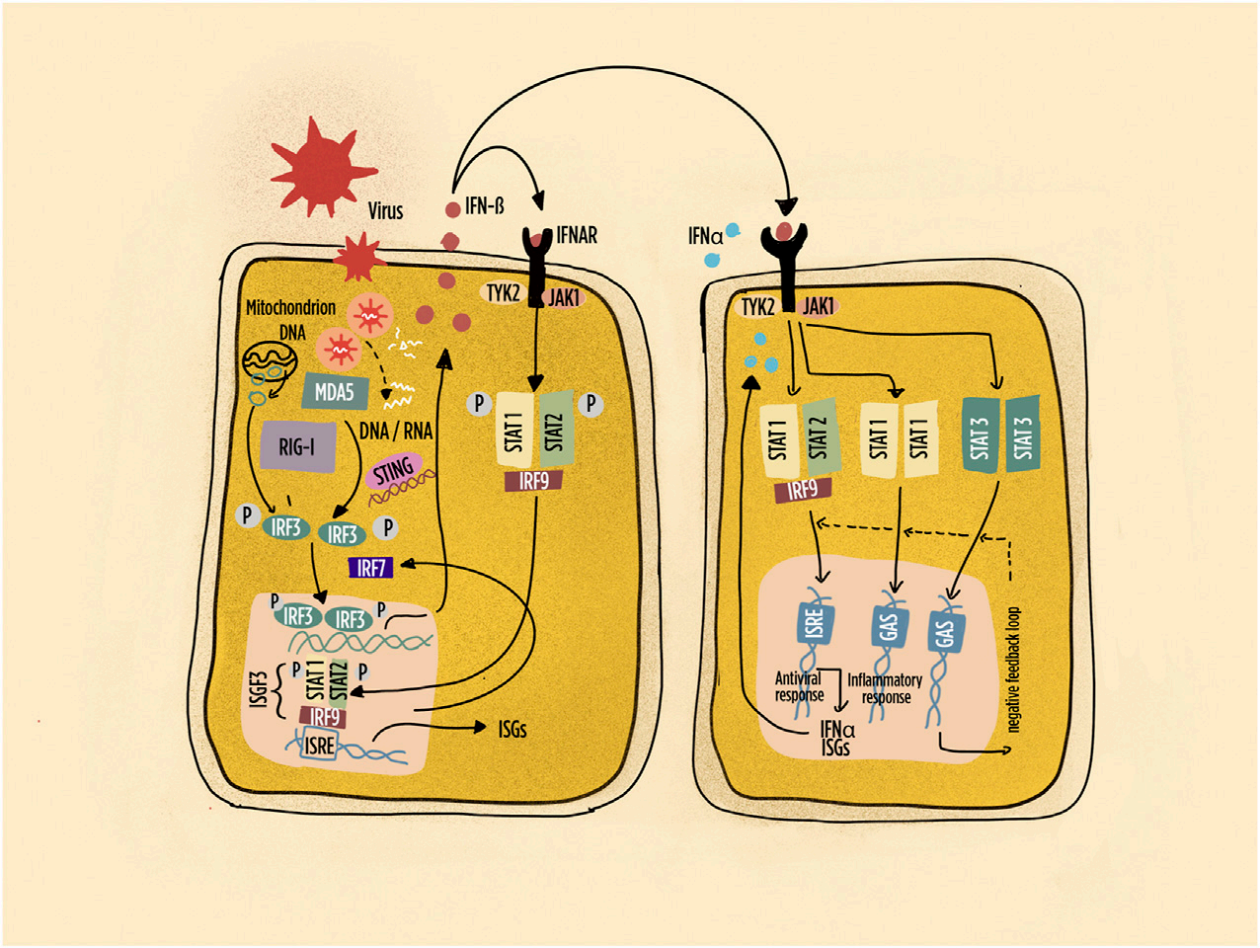
Schematic representation of pathways leading to interferon (IFN) production and IFN responses in many cell types. Highlighted the intracellular sensors of viral and endogenous DNA/RNA; the main interferon regulatory factors; the primary response characterized by IFN-beta production with autocrine and paracrine responses. Abbreviations: IFNAR: interferon-alpha/beta receptor; ISGF: interferon-stimulated gene factor; ISRE: interferon-stimulated response element; IRF: interferon regulatory factor; JAK: Janus kinase; MDA5: melanoma differentiation–associated protein 5; RIG-1: retinoic acid inducible gene-1; STAT: signal transducer and activator of transcription; STING: stimulator of interferon genes; Tyk: tyrosine kinase. Dashed line: negative feedback response.

The interferon (IFN) response indicates a chain of molecular events in cells and tissues which comprises identification of genetic material by pattern recognition receptors (PRRs), signal transduction and initiation of IFN production, the response to IFN, and expression of IFN-stimulated genes which then exert their function and establish regulatory feed-forward loops (Figure 1). IFNs have been originally described in 1957 as substances interfering with viral replication (Isaacs and Lindenmann, 1957). Since then, a large body of data implicates IFN in responses to viral infections by direct activities on infected cells and by profoundly influencing the behavior of cells of innate and adaptive immune response (Biron, 2001). Beyond antiviral activities, IFNs are involved in several biological processes playing a role in infectious diseases, cancer, inflammation, and autoimmunity. IFNs comprise a quite large family of proteins currently subdivided into type I IFN (IFN-I) including alpha (encoded by 13 distinct genes), -beta, -delta, -epsilon, -kappa and -omega families produced by almost all nucleated cell types and type III IFN (IFN-III) or IFN-lambda particularly produced by cells of hematopoietic origin and by epithelia at barrier surfaces. In contrast, the production of type II IFN (IFN-II) or immune interferon or interferon gamma is largely restricted to cells of lymphoid origin, particularly NK and T cells. All IFN-I signal through an invariant two-chain receptor expressed on most cell types. Similarly, IFN-II uses a dedicated two-chain receptor also expressed on most cell types. At variance, the IFN-III two-chain receptor is preferentially expressed on cells of epithelial origin and on plasmacytoid dendritic cells (pDCs). The three sets of IFN receptors converge toward the JAK/STAT (Janus kinase/signal transducer and activator of transcription) signaling pathways, which may account to some extent for the partially overlapping sets of genes activated in response to distinct IFNs (Hertzog et al., 2011; Weerd and Nguyen, 2012) (Table 1; Figure 2). While IFN-I and IFN-III ISG repertoires generally overlap, IFN-III signaling leads to a more sustained expression of ISGs, and in contrast to IFN-I, IFN-III does not induce the transcription of pro-inflammatory cytokines (Galani et al., 2017; Lazear et al., 2019).

**TABLE 1:** Receptors and main signaling molecules used by IFNs.

	IFN-I	IFN-II	IFN-III
Receptor subunits	IFNAR1	IFNGR1	IFNLR1
	IFNAR2	IFNGR2	IL-10R2
Receptor expression	All nucleated cells	All nucleated cells	Epithelial cell pDCs
Signaling molecules	JAK1 and TYK2	JAK1 and JAK2	JAK1 and TYK2
Transcription factors	STAT1/STAT2/IRF9	STAT1/STAT1	STAT1/STAT2/IRF9
	STAT1/STAT1		STAT1/STAT1

Evidence linking IFN to autoimmunity was published first in 1969 when poly I:C injection, which in a sense mimics viral infection, was shown to enhance disease manifestations in the (NZB/NZW) F1 murine lupus model (Steinberg et al., 1969). Thereafter, increased levels of IFN biological activity were documented in the serum of individuals suffering from various systemic autoimmune diseases (SADs) including systemic lupus erythematosus (SLE), rheumatoid arthritis (RA), systemic sclerosis (SSc), and Sjogren’s syndrome (SS) (Skurkovich et al., 1977; Hooks et al., 1979; Ytterberg and Schnitzer, 1982). In the following 40 years, it has been solidly established that systemic autoimmunity beyond SLE, RA, SSc, and SS to include myositis, mixed connective tissue disease (MCTD), and undifferentiated connective tissue disease (UCTD) is associated with conspicuous IFN biological activities (Higgs et al., 2011; Ekholm et al., 2016; Barturen et al., 2020). Further interest in IFN and SADs has spurred form the recent identification of monogenic disorders named interferonopathies characterized by high IFN production and clinical manifestations partly resembling those of SADs (Crow and Manel, 2015).

While IFNs are associated with SADs, the presence of high levels of IFNs is detectable in some but not all individuals suffering of SADs with frequencies of individuals with high IFN varying across the various SADs (Barturen et al., 2020). In the perspective of precision medicine, the identification of factors associated with high IFN may provide stratification of patients in order to offer them the most appropriate therapy. Excellent exhaustive reviews on the role of IFNs in SADs have been published in the last decade (Hall and Rosen, 2010; Ivashkiv and Donlin, 2014; Muskardin and Niewold, 2018; Crow et al., 2019; Rönnblom and Leonard, 2019). The present review will address succinctly several aspects linked with the role of IFNs in SADs taking SLE as prototypic for this class of diseases (Crow and Ronnblom, 2019). A particular attention will be devoted to mechanisms initiating IFNs production—which we name afferent function—and the role of IFNs in tissue pathology—to which we refer as efferent function. We will highlight the role of autoantibodies (autoAbs), immune complexes (ICs), and polymorphonuclear neutrophils (PMNs) in driving IFN production. While most of our attention will be dedicated to IFN-I, particularly to IFN-alpha, we will also review some aspects of IFN-II in SADs. Besides IFNs, many other factors play major roles in SADs with varying clinical and pathogenic associations (Kunz and Ibrahim, 2009; Simon et al., 2021). The complex mosaic of intervening cytokines is depicted in Supplementary Figure S1. Their description goes beyond the scope of the present review.

## What are the Mechanisms Leading to High IFN-I in SADs?

### IFN-I Producing Cells

**FIGURE 3:**
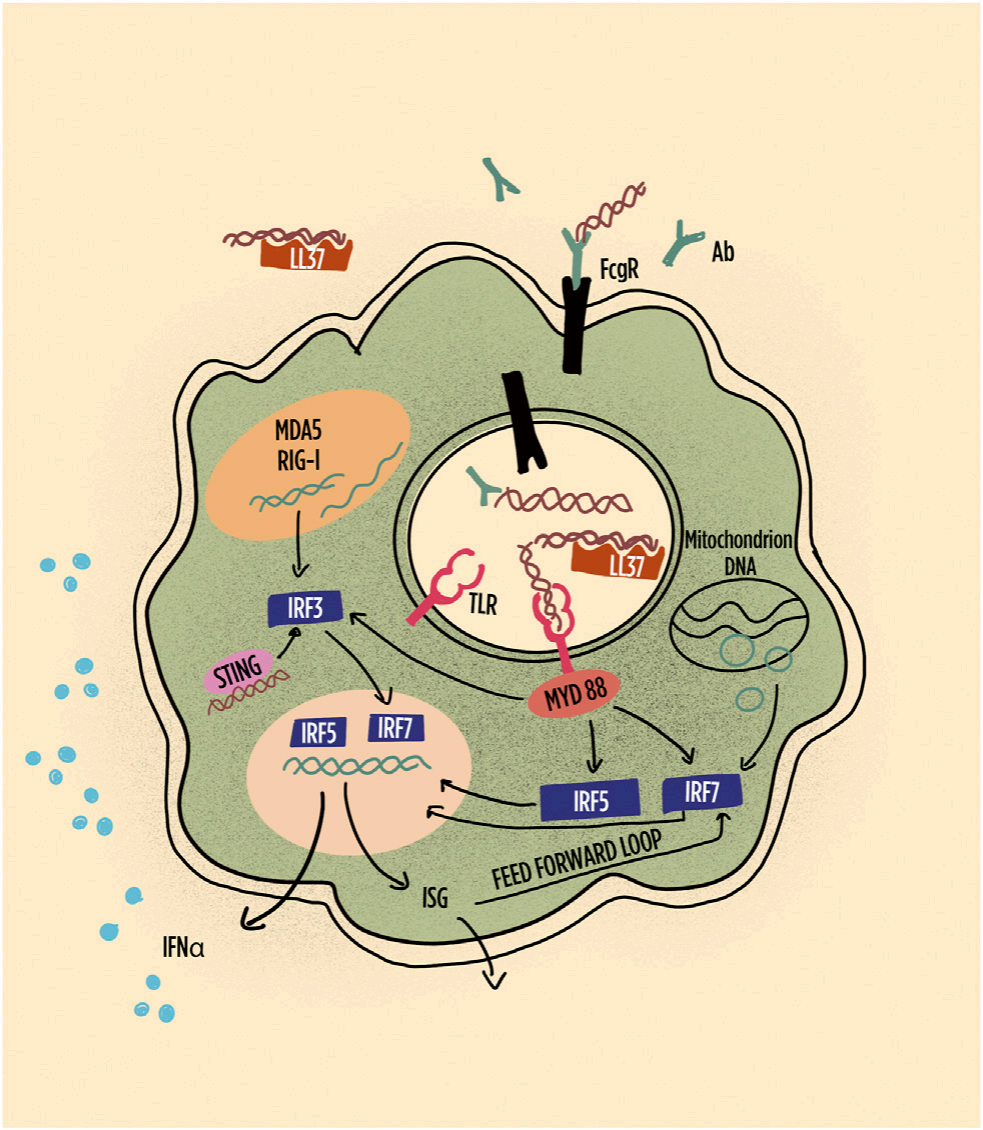
Schematic representation of pathways leading to interferon (IFN) production and IFN responses in plasmacytoid dendritic cells (pDCs). Highlighted are the role of immune complexes and LL-37 in shuttling DNA/RNA into endosomes; the subsequent the role of TLR in inducing interferon regulatory factors and their role in gene transcription of IFN-alpha and other interferon-induced gene products, including feed-forward loops. RNA, particularly long double-stranded RNA, is preferentially sensed by MDA5 and RIG1. Abbreviations: Ab: antibody; FcgR: Fc gamma receptor; IRF: interferon regulatory factor; LL-37: cathelicidin-37; MDA5: melanoma differentiation–associated protein 5; MyD88: myeloid differentiation primary response 88; RIG-1: retinoic acid inducible gene-1; STING: stimulator of interferon genes; TLR: toll-like receptor.

While almost all nucleated cells produce IFN-I including circulating leukocytes, pDCs expressing at their surface the inhibitory type II lectin receptor BDCA2 (blood dendritic cell antigen 2) are particularly potent and well-recognized producers of IFN-alpha (Rönnblom and Alm, 2001) (Figure 3). Of note, pDCs have been described infiltrating target organs in practically all SADs, thus reinforcing both the importance of IFN-alpha and of pDCs in immunopathology. Consistently with a central role of pDC in SLE, a recent phase 2 therapeutic trial assessing a monoclonal Ab ligating BDCA2 that inhibits the production of IFN-I and other inflammatory mediators has shown efficacy in reducing skin lesions and IFN signature in the blood (Furie et al., 2019a). PMNs have also been implicated in the production of IFN (Decker, 2011), particularly a subset named low density granulocyte (LDG) (Denny et al., 2010). PMN and LDG may participate to the IFN signature determined in peripheral blood and in tissue target of pathology (Kegerreis et al., 2019). This respect is relevant to stress that several cell types may contribute differentially to the production and IFN gene signature detected in blood. Thus, their relative numbers may affect the type and amount of detectable ISG. Keratinocytes and other epithelial cells are poised to respond and to produce IFNs. Characteristically, they produce IFN-III, but in addition, keratinocytes are high producers of IFN-kappa. Expression of interferon-kappa is significantly enhanced in keratinocytes upon viral infection, upon exposure to double-stranded RNA, or upon treatment with either interferon-gamma or interferon-beta (LaFleur et al., 2001). Most importantly, a very recent article reported primary production of IFN-kappa by keratinocytes in preclinical autoimmunity and SLE, simultaneously providing evidence for a functional impairment of pDC with defective production of IFN-α (Psarras et al., 2020). Consistently with these findings, when explored at single cell level by RNAsec, peripheral blood pDCs in SLE were found unable to produce IFN-α (Nehar-Belaid et al., 2020). These controversial findings highlight current difficulties in identifying the cells producing IFN-I in SADs.

### Methods Used to Detect IFN-I

It has to be taken in mind that in most instances is not IFN *per se* that has been detected but rather the expression of genes that are induced by IFN and referred to as interferon-stimulated genes (ISG) or IFN gene signature. This approach reflects the span of biological processes initiated and maintained by IFNs with hundreds of genes activated in cascade. Potentially, 10% of our genome may respond to IFNs (Schoggins, 2019). The IFN signature overcomes the technical difficulty to detect low levels of the various IFN class members by solid phase assays that have low sensitivity and, the detection of IFN biological activity, which while possessing higher sensitivity, requires cumbersome procedures. Nonetheless, the drawback of using ISG as readout for IFN production is linked to the partial overlap in the genes induced by the three classes of IFN, which may confound and complicate the interpretation of the data generated (Hall et al., 2012). Furthermore, under certain circumstances, sustained expression of a subset of ISGs can take place over prolonged time periods, even in the absence of ongoing cytokine-mediated signaling (Cheon et al., 2013). A more recent methodology named SIMOA (single molecule array) based on the paramagnetic detection of single molecules complexed on beads has been used to detect IFN-alpha with a sensitivity in the femtogram per ml range (Wilson et al., 2016).

### Mechanisms at Play in the Induction of IFN-I

Given the presence of IFNs in SADs, then the question arises about the mechanisms leading to IFN production in these pathological conditions. Type I and III IFN are physiologically produced when the presence of genetic material (DNA and RNA) of pathogen origin is sensed by specific receptors in the cytosol or in endosomes. However, also “self” DNA and RNA may activate such receptors when delivered in the appropriate manner (Barrat et al., 2005; Barrat et al., 2016; de Jong et al., 2016). Defective clearance of cells undergoing apoptosis or necrosis may provide the antigenic material composed of nucleic acids and nucleoproteins (Casciola-Rosen et al., 1994; Mahajan et al., 2016). Along the same line of evidence, polymorphisms of gene coding for enzymes deputed to DNA and RNA degradation are associated with an increased risk of SLE (Crow and Ronnblom, 2019). Some examples are polymorphisms of deoxyribonuclease 1 like 3 (*DNase1L3*), three prime repair exonuclease 1 (*TREX1*), SAM and HD domain containing deoxynucleoside triphosphate triphosphohydrolase 1 (*SAMHD1*), and ribonuclease H2 subunit A (*RNASEH2A*) (Jiang et al., 2020). Of interest, the loss of function of these very same genes is associated with interferonopathies, stressing the role of nucleic acid in the induction of IFN-I (Rodero and Crow, 2016). Well-identified receptors for the genetic material are TLRs (toll-like receptors) anchored on cell membranes or more specifically on internal organelle membranes. Additionally, cytosolic receptors play important roles in recognizing nucleic acids. They include RIG-1 (retinoic acid inducible gene-1), MDA5 (melanoma differentiation–associated protein 5), NLR (nucleotide oligomerization-like domain receptor), and cGAS–STING (cyclic GMP-AMP synthase-stimulator of interferon genes) (Crow et al., 2019) (Table 2). IFN-II is produced in response to the immune activation of NK and T cells, with IFN-I, IFN-II, interleukin-12 (IL-12), and IL-18 playing a major role in the induction of IFN-II.

**TABLE 2:** Main signaling pathways leading to IFN-I production.

Ligand	Receptor	Proximal transducing molecule	Transcription factors^a^
Extracellular DNA	TLR9^b^	MyD88	IRF5 and IRF7
Extracellular RNA	TLR7^b^	MyD88	IRF5 and IRF7
Extracellular dsRNA	TLR3^b^	TRIF	IRF3 and IRF7
Intracellular long dsRNA	MDA-5	MAVS	IRF3, IRF5, and IRF7
Intracellular short dsRNA	RIG-1	MAVS	IRF3, IRF5, and IRF7
ssRNA	NLR^c^		IRF3 and IRF5
Intracellular DNA	cGAS	STING	IRF3, IRF5, and IRF7

^a^ These transcription factors work in concert with additional molecules forming transcriptional complexes.

^b^ Present in endosomes. The ligands need to be shuttled into endosomes to activate the receptors.

^c^ NLRs comprise three families of proteins: NOD (NOD1-2, NOD3/NLRC3, NOD4/NLRC5, NOD5/NLRX1, and CIITA), NLRP (NLRP1-14, also referred to as NALP), and IPAF.; Abbreviations: cGAS: cGMP-AMP synthase; CIITA: class II, major histocompatibility complex, transactivator; IRF: interferon regulatory factor; MDA5: melanoma differentiation–associated protein 5; MAVS: mitochondrial antiviral signaling protein; MyD88: myeloma differentiation primary response 88; NLRs: nucleotide-binding oligomerization domain-like receptors; NLRP: nucleotide-binding oligomerization domain, leucine-rich repeat and pyrin domain containing (also abbreviated as NALP); NOD: nucleotide oligomerization domain; RIG-1: retinoic acid inducible gene-1; STING: stimulator of interferon genes; TLR: toll-like receptor; TRIF: TIR-domain–containing adapter-inducing IFN-beta. Primary data from (Zhao et al., 2015; Zarrin et al., 2020).

Immune complexes (ICs) containing DNA or RNA, eventually associated with nucleoproteins, are well documented and important inducers of IFN-I in SADs. Defective clearance of apoptotic material may provide the antigenic material targeted by autoAb forming these ICs (Casciola-Rosen et al., 1994). Defective digestion of extracellular genetic material may enhance this phenomenon (Sisirak et al., 2016). In these ICs, autoAbs are captured by FCgamma receptors at the cell surface and shuttled with their antigen in the endosomal compartment where they activate TLR7 (RNA) or TLR9 (DNA). This was initially demonstrated in SLE, SS, and SSc by Ronnblom and colleagues (Vallin et al., 1999; Bave et al., 2000; Bave et al., 2005; Kim et al., 2008). Furthermore, IFN-I in SLE serum was shown to capacitate the maturation of DCs from monocytes (Blanco et al., 2001), and DCs are fundamental to activate T cells and could favor the resurgence of autoreactive T cell clones which may at their turn provide appropriate help to autoreactive B cells. This may therefore link the enhanced production of IFN-I to adaptive immunity. However, the autoAbs recognizing self-DNA or RNA are themselves the product of adaptive immunity, which therefore should have preceded the formation of immune complexes. Thus, ICs potently enhance IFN-I production and participate to an amplification loop resulting in higher IFN-I production. A question that has not been addressed formally yet is whether autoAbs are needed for an IFN-I initial production. A corollary of this question is whether natural Abs, which are produced in the absence of antigenic stimulation and have low affinity and are polyreactive, may appropriately present self-nucleic acids to IFN-I producing cells (Madi et al., 2012).

A more recently described mechanism shown to favor the production of IFN-I by pDC involves the capacity of amphipathic peptides to form complexes with extracellular DNA or RNA allowing the shuttling of this material into endosomes (Lande et al., 2007; Ganguly et al., 2009; Lande et al., 2019). When properly directed into endosomes, the genetic material activates TLR7, TLR8, or TLR9. Notably, amphipathic peptides deliver DNA or RNA into endosomes as efficiently if not more compared to autoAb (Lande et al., 2019). Examples of these peptides are, among others, cathelicidin also known as LL-37 and chemokines such as CXCL4 also known as platelet factor 4 (PF4). LL-37 is produced mainly by keratinocytes and PMN, both important players in SLE pathogenesis. PF4, abundantly released by platelets, is produced by several cell types of hematopoietic origin. Most interesting, LL-37 decorates DNA when extruded by PMN forming extracellular traps (NETosis) (Kahlenberg and Kaplan, 2013). This active process characterizes SLE and many other SADs (Granger et al., 2019). Thus, PMNs undergoing NETosis provide both DNA and peptides that favor its entry in pDC resulting in enhanced IFN-I production (Lande et al., 2011). In the perspective of the present review, the question then arises whether in SLE and in other SADs the propensity of PMN to participate to disease pathogenesis is a primary or a secondary event (Wirestam et al., 2019). In other words, whether the activation and subsequent NETs formation by PMNs, which favor IFN-I production, is intrinsic to PMNs or whether they become activated because other pathogenic phenomena occur. For instance, it is well described that immune complexes have the capacity to activate PMNs which then undergo NETs formation (Lande et al., 2011). Furthermore, both LL-37 and CXCL4 were shown to be target of autoAbs that participate to both enhanced NETosis and enhanced stimulation of pDC to produce IFN-I. Indeed, genes associated with granulopoiesis were shown to be expressed in active SLE (Bennett et al., 2003) and characterize a subgroup of pediatric SLE (Banchereau et al., 2016), a robust finding confirmed when adult and pediatric SLE cohorts were pooled for the analysis (Toro-Dominguez et al., 2018). Both in SLE and in RA (Wright et al., 2017), low-density granulocytes (LDGs) have been identified which may represent a distinct cell population with specific functions. In SLE, but not in RA, the majority of LDGs, namely, the LDG expressing CD10, do not appear to be immature cells, but rather activated cells with enhanced expression of ISG, and enhanced function including enhanced NETosis, degranulation, chemotaxis, and release of oxidized mitochondrial DNA (Mistry et al., 2019). In SLE, CD10pos LDGs share with classical PMN enhanced expression of ISG. ISGs are not activated in immature CD10neg LDG (Mistry et al., 2019). Thus, a subset of LDG in SLE may represent an intrinsically pathogenic cell type, although this remains to be demonstrated. However, their number correlates with disease activity and organ damage (Mistry et al., 2019).

Several additional mechanisms may account for enhanced IFN-I production, in particular by pDCs. Briefly, they include activation by T cells, NK cells, B cells, and platelets each with a dedicated combination of signaling molecules (Rönnblom and Leonard, 2019). Recently, attention has been given to the capacity of mitochondrial DNA and long interspersed element 1, belonging to transposable DNA elements, to stimulate IFN-I production by exploiting cytosolic DNA sensors (Lood et al., 2016; Mavragani et al., 2016).

## What are the Predisposing Conditions Favoring High IFN-I Production?

### Gene Polymorphisms Modulating IFN-I Production and IFN Responses

Several gene polymorphisms associated with an increased risk of disease development are shared between SADs (Teruel and Alarcón-Riquelme, 2016). Of interest, many of them are localized in the IFN pathway (Jiang et al., 2020). These polymorphisms may modulate IFN production when signals are propagated.

### IRF5

The signaling cascade initiated among others by TLR7 and TLR9 occupancy leads to IRF5 phosphorylation, homo- or heterodimerization, nuclear translocation, and binding to the promoters of type I IFN genes (Figure 3). IRF5 polymorphisms have been associated with SLE and several other SADs (Sigurdsson et al., 2005; Graham et al., 2007; Dieude et al., 2009; Miceli-Richard et al., 2009; Tang et al., 2014; Matta and Barnes, 2020). Consistently with the role of IRF5 and IFN-I in SLE, the lack of IRF5 prevents disease development in models of murine lupus (Richez et al., 2010). In SLE, enhanced levels of serum IFN-I have been associated with IRF5 polymorphisms (Niewold et al., 2008). Interestingly, however, high levels of IFN-I, assessed by a functional assay, were not uniformly distributed according to IRF5 polymorphisms. Rather different IRF5 haplotypes characterized by a different combination of functional genetic elements were associated or with anti–double-stranded DNA (dsDNA) or with anti-SSA/Ro52 autoAb (Niewold et al., 2012). Thus, the genetic risk was revealed by the presence of patient-restricted autoantibodies.

### IFIH1

Similar findings were obtained when assessing the association between a polymorphism of IFIH1 (IFN induced with helicase C domain 1; also known as MDA5) and IFN-I serum levels in SLE (Robinson et al., 2011). IFIH1 is a cytoplasmic dsRNA sensor that activates IFN-α pathway signaling. In this case again, higher serum IFN-I levels were detected only in individuals with the appropriate allele and positive for DNA autoantibodies. We share with the authors the opinion that these data support a model in which autoAb and the formation of immune complexes then lead to enhanced IFN-I production. These data are consistent with the contention that adaptive immune responses precede enhanced production of IFN-I.

### STAT4

STAT4 is activated downstream of a number of cytokines, including type I IFNs and contributes to T cell differentiation and IFN-γ production. Polymorphisms within STAT4 have been linked with an increased risk of RA, SLE, SSc, and SS (Remmers et al., 2007; Dieude et al., 2009; Nordmark et al., 2009; Gestermann et al., 2010). Furthermore, patients with SLE and the STAT4 risk haplotype have a more severe disease phenotype (Taylor et al., 2008). Of interest, a STAT4 variant (T allele; rs7574865) was reported to render SLE peripheral blood mononuclear cells more responsive to IFN-I as assessed by their expression of ISG (Kariuki et al., 2009). Further, the same variant was associated with enhanced production of IFN-gamma by CD4 and CD8 T cells in response to both IFN-I and IL-12 (Hagberg et al., 2018). These data provide evidence that polymorphisms downstream IFN-I signaling, therefore belonging to their efferent function, have functional consequences. Interestingly, they appear to participate to enhance IFN-II production.

### Downstream Effects and Regulation of IFN-I Signaling

The first consequence of IFN-I production is an enhanced production of IFN-I itself (Figure 1). This feed-forward loop, elegantly discussed by John C. Hall and Antony Rosen (Hall and Rosen, 2010), results in rapid amplification of IFN-I responses. Mechanistically, many of the receptors, which include cytoplasmic and endosome sensors of nucleic acids, signal transduction molecules and transcription factors that drive IFN production are themselves regulated by IFNs. These feed-forward loops favor the expression of hundreds of genes and create the potential for amplifying immunopathology in SADs by affecting the function of target cells in the tissues and by modulating the activity of antigen presenting cells and effector cells of the immune system (Biron, 2001). Upon the initial induction of IFN-I production, remarkable are the explosive production of IFN-I by pDC and antigen presentation by conventional DC, including the expression of co-stimulatory molecules and the activation of potentially autoreactive T and B cells. Further, several autoantigen targets of autoAb are highly responsive to IFN, potentially augmenting antigen drive in SADs. Among others, the expression of the autoantigen SSA/Ro 52 kD, which has direct antiviral properties, increases in cells under the influence of IFN-I enhancing the presentation of immunostimulatory Ro52 epitopes (Strandberg et al., 2008). Indeed, the autoantigen SSA/Ro 52 is targeted by autoAb in several SADs, with the titer of anti-SSA/Ro autoAb remaining stable during disease evolution.

IRF3 activation and IFN-β production are observed in most cells at the initiation phase of the IFN-I response (Figure 2). The autocrine and paracrine activity of IFN-β then induces the expression of IRF7, which positively regulates IFN-I production in adjacent cells. Of interest, pDCs constitutively express high levels of IRF7, thus explaining their rapidly and potent response to activation. In addition, pDCs express IRF5, which induces the transcription of IFN-α genes distinct from those induced by IRF7 (Barnes et al., 2004). Indeed, pDCs have been identified in target organs of virtually all SADs, and IRFs are critical regulators of the quality and quantity of IFN-I responses (Jensen and Niewold, 2015; Ye et al., 2020). The response to IFNs, mediated by the canonical JAK-STAT signaling transduction pathway, is further modulated by the composition of the molecular complexes involved in gene transcription (Table 1). Thus, the interferon-stimulated gene factor 3 (ISGF3) complex (composed of STAT1, STAT2, and IRF9) activates classic antiviral genes. By contrast, STAT1 homodimers induce pro-inflammatory gene expression, and STAT3 homodimers suppress pro-inflammatory gene expression (Ivashkiv and Donlin, 2014), participating to downregulating regulatory loops. Additional complexity is provided by the contribution of signaling pathways involving MAPK (mitogen-activated protein kinase), NFkappaB (nuclear factor kappa-light-chain-enhancer of activated B cells), and PKB (protein kinase B) which may influence the composition of ISGF and the consequent activation of specific genes triggered by IFN-I. Host factors such as the concurrent presence of inflammatory cytokines or chemokines therefore participate to the modulation both positive and negative of IFN-I signaling (Ivashkiv and Donlin, 2014). It is worth to stress here that IFN-I and TNF tend to cross talk resulting in reciprocal inhibition (Banchereau et al., 2004). Indeed, therapies based on TNF blockade may result in enhanced expression of ISG in the peripheral blood (Mavragani et al., 2007), a mechanism potentially at play in TNF blockade–induced SLE. It is therefore remarkable that the tyrosine-protein phosphatase non-receptor type 22 (PTPN22) C1858T polymorphism is associated with skewing of cytokine profiles toward high IFN-I activity and low TNF levels in patients with SLE (Kariuki et al., 2008). However, in synovial RA macrophages, TNF drives ISG expression, but at the same time, it limits type I IFN-mediated signaling and modulates the pattern of ISG expression (Gordon et al., 2012). Further, TNF may participate to IFN-beta induction by IRF1 signaling (Yarilina et al., 2008).

Overall, the different IFN types and subtypes participating to responses and the cell intrinsic and distinct temporal distribution of molecular complexes involved in IFN intracellular signaling concur in modulating their effects on the quality and quantity of gene transcribed, which may account for the IFN heterogeneous biological and pathological effects.

## What is the Role of IFN-I in the Development of Distinct Clinical Manifestations Within SADs?

### Systemic Autoimmune Diseases Heterogeneity and IFN-I

Clinical manifestations and biological abnormalities allow to distinguish between SADs and classification criteria perform well enough to group patients with diverse and different disease manifestations. To a large extent, these differences lead to different therapeutic strategies applied to SADs. This notwithstanding, a common set of 36 type I IFN inducible transcripts was identified among the most overexpressed in the whole blood of 262 patients with SLE, RA, SSc, and myositis in contrast to 26 healthy controls (Higgs et al., 2011). Along the same line of evidence, when unsupervised clustering of integrated whole blood transcriptome and methylome was performed with data of 263 healthy controls and 918 patients with seven SADs (SLE, RA, SS, SSc, MCTD, antiphospholipid syndrome, and UCTD), among the four identified clusters, the “interferon” cluster was grouping individuals with all seven distinct SADs (Barturen et al., 2020). Thus, an IFN signature is common to all SADs, which associated with the genetic polymorphisms of IFN-related genes shared between SADs, may account for part of the “heritability” of systemic autoimmunity (Niewold et al., 2007; Kariuki et al., 2010). Beyond this commonality, subtler analyses may provide important information accommodating heterogeneity in clinical manifestations between and within SADs. In this respect, the subdivision of ISG modules based on complex correlations and factor analysis within expressed genes resulted in two simplified IFN scores that allowed categorization of SLE vs. RA (El-Sherbiny et al., 2018).

*Systemic lupus erythematosus*. Therapeutic use of IFN-α, for instance, in the setting of chronic hepatitis C infection, may lead to clinically overt SLE, which regresses after therapy suspension (Niewold and Swedler, 2005). Consistently with a pathogenic role, a rise in circulating IFN-I precedes disease manifestations in SLE and accompanies disease severity (Bengtsson et al., 2000; Baechler et al., 2003; Kirou et al., 2005; Munroe et al., 2016). However, an IFN signature is found in only 50–80% of SLE patients (Baechler et al., 2003; Bennett et al., 2003; Crow and Wohlgemuth, 2003) and the IFN-induced gene signature assessed in longitudinal studies may not correlate with disease activity (Landolt-Marticorena et al., 2009; Petri et al., 2009). Indeed, in addition to IFN modules, other gene modules have been variably reported to associate with SLE clinical features (Banchereau et al., 2016; Rai et al., 2016; Lu et al., 2019; Barturen et al., 2020; Guthridge et al., 2020). These data indicate that IFN does not account for all pathological and clinical aspects of SLE, which may be further explained by heterogeneity in the IFN-I pathway activation and genetic makeup (Kariuki et al., 2015). In a pediatric SLE population undergoing frequent relapses, the modules of IFN-I-related genes were among the most prevalent with those related to the myeloid lineage (Banchereau et al., 2016). In accordance with others, we found that in multivariate analysis, only mucocutaneous and articular SLE clinical manifestations were specifically associated with high IFN-I gene signature detected in peripheral blood (Chasset et al., 2020). Of interest, the clinically most active patients combined higher expression of IFN-I and PMN genes in peripheral blood (Chasset et al., 2020). To be noted, however, that the risk of relapse appears to increase in SLE patients with high vs. low IFN-alpha levels, when assessed by SIMOA (Mathian et al., 2019). In addition, when assessing gene expression, a relationship between inactive vs. moderately active or very active disease was found with diverse modules of expressed genes, with a contribution by IFN-beta and IFN-gamma in addition to IFN-alpha (Jourde-Chiche et al., 2016). Similarly, high levels of circulating interferons type I, type II, and type III were found to be associated with distinct clinical features of active SLE (Oke et al., 2019). IFN-kappa expressed in keratinocytes and *IFNk* gene polymorphisms in SLE appear to be involved in cutaneous manifestations accelerating responsiveness of epithelia to IFN-α and increasing keratinocyte sensitivity to UV irradiation. (Harley et al., 2010; Sarkar et al., 2018). IFN-III also appears to have a role in SLE skin lesions (Zahn et al., 2011). “Natural autoantibodies” directed against IFN-alpha have been reported in SLE positively correlating with disease activity (Gupta et al., 2016). However, a subset of these were blocking autoAb and were associated with the absence of IFN gene signature and reduced SLE disease activity (Gupta et al., 2016). Of great interest, in a longitudinal study addressing the presence of IFN in the sera of individuals which would develop SLE, the presence of IFN-II and of chemokines induced by IFN-II temporally preceded the detection of IFN-I itself associated to the increased presence of autoAb directed against nucleoproteins or DNA. The clinical manifestations then followed (Munroe et al., 2016). Within the limits of the relatively low number of individual tested and the sensitivity of the assays used to detect IFN-I and IFN-II, this is an important piece of evidence indicating that an adaptive immune response in SLE precedes and accompanies the initial detection of IFN-I (Lu et al., 2016). Along the same line of evidence, clinical responders, as opposed to nonresponders in a phase 2 trial assessing the efficacy and safety of ustekinumab (anti-IL-12/IL-23) in SLE had treatment-dependent reduced serum levels of IFN-gamma and not of other cytokines (van Vollenhoven et al., 2018; Cesaroni et al., 2020; van Vollenhoven et al., 2020).

*Sjögren syndrome*. SS classical clinical manifestations include dry eye and dry mouth due to exocrine gland inflammation. However, systemic manifestations are frequent with involvement, among others, of the peripheral nervous system, skin, and kidneys distinctly different from those observed in SLE. However, SS very much resemble SLE in terms of IFN-I signature detected in peripheral blood and shared genetic risk factors. Thus, in SS, a peripheral blood IFN-I gene signature strongly correlates with the presence of anti-SSA/Ro 52 autoAb (Emamian et al., 2009). Of interest, studies of minor salivary glands revealed both enhanced IFN-I and IFN-II gene signatures with IFN-II being predominant and associated with lymphomagenesis risk (Nezos et al., 2015). In addition, in salivary glands, epithelial cells were contributing to IFN-beta and infiltrating pDC to IFN-alpha production (Mavragani et al., 2016).

*Myositis*. Primary inflammatory myositis comprise a large array of distinct clinical syndromes in which muscle inflammation is often accompanied by skin, joint, lung, vascular, and other abnormalities. Perhaps, myositis as a whole is the SAD with the best correlation between IFN-I levels and disease activity. In myositis, IFN-I levels are increased in the circulation and most interestingly in muscle tissue with an association with disease activity (Niewold et al., 2009; Greenberg et al., 2012). Along the same line of evidence, pharmacological inhibition of JAK signaling in a clinical trial improved IFN-I–induced muscle fiber damage (Ladislau et al., 2018). Studies of muscle biopsy showed that immature muscle precursor cells overexpressing HLA class I are a source of IFN-beta, which may play a direct role in the induction of IFN-I signature in muscle fibers (Tournadre et al., 2012). Serum IFN-beta rather than IFN-alpha levels appear to correlate with cutaneous manifestations and their severity in dermatomyositis (Huard et al., 2017). Further, IFN-I enhances the expression of some autoantigens including MDA5 which defines a very specific clinical subtype of dermatomyositis, thus reinforcing a pathogenic role of IFN-I in myositis subsets (Sato et al., 2009; Fiorentino et al., 2011).

*Systemic sclerosis*. SSc is characterized by fibrosis of the skin and internal organs including the lung, the gastrointestinal tract, and heart, accompanied by prominent vasculopathy. SSc shares with the other SADs, and with SLE in particular, both high IFN-I gene signature in peripheral blood (Assassi et al., 2010) and gene polymorphisms of the IFN pathway linked to increased risk of disease. Of pathogenic interest, the IFN-I gene signature may precede the development of lung fibrosis (Brkic et al., 2016). Peculiar to SSc, the very high serum levels of CXCL4, which are associated to lung fibrosis and pulmonary arterial hypertension (van Bon et al., 2014). CXCL4 acts as a chaperone shuttling extracellular DNA into endosomes in pDC enhancing in TLR8- and TLR9-dependent manner the production of IFN-I (Ah Kioon et al., 2018; Lande et al., 2019).

*Rheumatoid arthritis*. RA is mainly characterized by erosive symmetrical arthritis, but systemic manifestations may involve the lung, the skin, and other organs. While the IFN-I signature is less conspicuous in RA than other SADs including SLE (Higgs et al., 2011; Barturen et al., 2020), an IFN-I signature precedes overt clinical manifestations and its presence increases the risk of developing the disease (Lübbers et al., 2013). The presence of pDCs and the expression of ISGs, IFN-alpha, and IFN-beta have been documented in the synovium of patients with RA (Lande et al., 2004; van Holten et al., 2005). Of interest, monocytes, chondrocytes, and fibroblast-like synoviocytes were shown to respond to IFN-beta by enhanced production of the anti-inflammatory of IL-1 receptor antagonist (Coclet-Ninin et al., 1997; Palmer et al., 2004), which may counteract the potentiation by IFN-alpha of the TLR4-mediated production of IL-1-beta in RA synovial cells (Roelofs et al., 2009). In relationship with these observations, two independent studies consistently reported that the response to tumor necrosis factor (TNF) inhibitors in RA was predicted by the ratio of pretreatment IFN-beta to IFN-α activity, with lower ratios (higher IFN-beta) associated with responses to TNF inhibition (Mavragani et al., 2010; Wampler Muskardin et al., 2016). To extend these findings, the ratio between IFN-alpha and IFN-beta appears to vary in different SADs, suggesting a complex participation of these two IFN subtypes to autoimmunity (de Jong et al., 2016).

## Would Therapeutic Strategies Targeting IFN-I be Helpful in Controlling or Even Preventing SADs?

Better understanding for the role of IFN-I in SADs has led to a wide array of therapeutic strategies aiming at blocking, neutralizing, and inhibiting IFN-I or inhibiting intracellular signaling initiated by IFN receptor engagement or targeting high IFN-I–producing cells (Chasset and Arnaud, 2018; Jiang et al., 2020). Since the proximal intracellular signaling pathways are shared by IFNs with several other cytokines, the expected inhibitory effects of intracellular signaling inhibition are broader than those resulting from direct IFN or IFN receptor inhibition (Kubo et al., 2018). Indeed, it has been estimated that more than 40 types of cytokines transmit signals through the JAK/STAT pathway (Kubo et al., 2019). On these bases, the inferences on the role of IFNs in SADs that can be deduced from therapeutic approaches will be dependent on the inhibition strategy and may vary substantially. Here, we provide a non-exhaustive overview of therapeutic approaches currently being tested in clinical trials, highlighting the differences between the various treatment strategies. The molecules under current testing in clinical trials having reached at least phase 2 levels are reported in Table 3.

**TABLE 3:** Clinical trials of molecules targeting IFN-I, cells producing IFN-I, or IFN-I–related signaling pathways in clinical development ≥ phase two trials in lupus erythematosus.

Type of inhibitor	Name	Current developmental phase	Primary outcome achieved	Main outcome	Refs
Anti–IFN-α mAb	Rontalizumab	Phase 2	No	BILAG at w24	Kalunian et al. (2016)
	Sifalimumab	Phase 2	Yes	SRI-4 at w52	Khamashta et al. (2016)
**Therapeutic vaccine IFN-α**	**Interferon-α-kinoid**	**Phase 2b, ongoing phase 3**	**No**	**Modified BICLA at w36**	Houssiau et al. (2020)
**Anti-IFNAR1 mAb**	**Anifrolumab**	**Phase 3**	**Yes/No**	**SRI-4/BICLA at w52**	Furie et al. (2019b); Morand et al., (2020)
**Anti-pDC**	**BIIB059**	**Phase 2, ongoing phase 3**	**Yes**	**% Change in CLASI-A at w16**	Furie et al. (2019a)
**JAK1/JAK2 inhibitors**	**Baricitinib**	**Phase 2, ongoing phase 3**	**Yes**	**Arthritis/rash SLEDAI-2K at w24**	Wallace et al. (2018)
**JAK1/JAK3 inhibitors**	**Tofacitinib**	**Ongoing phase 2 in CLE**	**NA**		
JAK1/JAK2/JAK3 inhibitor	Tanzisertib	Phase 2	No	NA	
JAK1 selective inhibitor	Solcitinib	Phase 2	No	NA	
	**Filgotinib**	**Phase 2 in CLE**	**No**	**% Change in CLASI-A at w12**	
Topical JAK/SYK inhibitor	R333	Phase 2	No	≥50% decrease CLASI-A at w4	Presto et al. (2018)
**Tyk-2 inhibitor**	**BMS-986165**	**Ongoing phase 2**	**NA**		
**Syk inhibitors**	**Lanraplenig**	**Phase 2**	**NA**		Blomgren et al. (2020)
	Fostamatinib	Phase 2	NA		
**BTK inhibitors**	**Evobrutinib**	**Phase 2**	**NA**		Haselmayer et al. (2019)
	**Elsubrutinib ABBV-105**	**Phase 2**	**NA**		Goess et al. (2019)
	**Fenebrutinib**	**Phase 2**	**No**	**NA**	Lorenzo-Vizcaya et al. (2020)
TLRs 7, 9 inhibitors	DV1179	Phase 2a	No	NA	

In bold, molecules currently in continuous clinical development. BTK: Bruton tyrosine kinase; IFN: interferon; IFNAR1: interferon-alpha receptor 1; JAK: Janus kinase; pDCs: plasmacytoid dendritic cells; SYK: spleen tyrosine kinase; TLR: toll-like receptor; Tyk 2: tyrosine kinase-2.

### Anti–IFN-Alpha Monoclonal Antibodies

The recombinant technology offers the possibility to raise high-affinity, neutralizing monoclonal antibodies (mAbs) against IFN-alpha. The specific difficulty here is linked to the fact that there are 13 different subtypes of IFN-alpha, with 75–99% amino acid sequence identity and different affinities for their receptor (Gibbert et al., 2013). While the mAbs which underwent clinical development were claimed to bind to and neutralize the majority of IFN-alpha subtypes, most likely they did not have the possibility to neutralize all the IFN-alpha biological activity. Further, this approach did not neutralize other type I IFNs which could have relevant pathological activities in SADs. However, interesting but somehow discouraging results were obtained in clinical trials in which anti–IFN-alpha mAbs were tested in myositis, SSc, and SLE. A phase 1b randomized, placebo controlled, clinical trial was conducted to evaluate sifalimumab (MEDI-545) in dermatomyositis (*n* = 27) and polymyositis (*n* = 21). Sifalimumab suppressed the type I IFN gene signature by 66% in the blood and 47% in the muscle at day 98. The authors reported a positive correlative trend between target neutralization and clinical improvement (Higgs et al., 2014), suggesting that direct type I IFN-I inhibition may be efficacious in myositis. To the best of our knowledge, however, no other clinical trials are currently conducted with this molecule in myositis. Rontalizumab and sifalimumab both reached phase 2 (Jiang et al., 2020) in clinical trials in SLE. Rontalizumab decreased the expression of ISG in phase 1 study with an acceptable safety profile (McBride et al., 2012). However, in the phase 2 study (*See* Supplementary Table S1 for definitions of BILAG and other terms), the efficacy response rates assessed by the British Isles Lupus Assessment Group (BILAG)–based Composite Lupus Assessment (BICLA) (primary endpoint) and the Systemic Lupus Erythematosus Responder Index (SRI)-4 at week 24 (secondary endpoint) were similar between rontalizumab and placebo, and its development was terminated (Kalunian et al., 2016). Sifalimumab, a fully humanized IgG1 kappa anti–IFN-α mAb demonstrated in a phase 2b study higher SRI-4 response index at week 52 than placebo. Of interest, sifalimumab efficacy was statistically significant in patients with high but not with low IFN-I gene expression signature, which hints to the advantage of selecting patients for this therapeutic approach (Khamashta et al., 2016).

### Interferon-α-Kinoid

An alternative strategy explored the potential of neutralizing IFN-I by eliciting an endogenous immune response against IFN-I. This requires to break tolerance against self-IFN and induce autoimmunity. It was achieved with a therapeutic vaccine named interferon-α-kinoid (IFN-kinoid) composed of IFN-α-2b coupled to a carrier protein containing T helper cell epitopes that induces polyclonal anti-IFN-α neutralizing antibodies. In a phase 1/2 study, IFN-kinoid–induced anti–IFN-α antibodies in all immunized patients and significantly reduced the expression of the IFN gene signature compared to placebo (Ducreux et al., 2016). In a phase IIb study, 91% of immunized individuals having received five doses of vaccine developed detectable neutralizing antibodies. Overall, the IFN high gene expression signature decreased by 31%, but in 20/87 individuals with low titers of anti–IFN-alpha Ab (20/87), the IFN gene signature actually increased during the trial period. In the whole population, modified BICLA responses at W36 did not statistically differ between IFN-kinoid (41%) and placebo (34%) (Houssiau et al., 2020). However, attainment of lupus low disease activity state (LLDAS) at W36 discriminated the two groups in favor of IFN-kinoid (53 vs. 30%, *p* = 0.0022) with a significant glucocorticoid sparing effect. These analyses restricted to the subgroup of individuals having developed detectable anti-IFN-alpha antibody were all statistically significant. Interestingly, the immune response elicited by IFN-kinoid was not restricted only to IFN-α-2b but encompassed with variable efficacy also other members of the IFN-alpha subfamily in 50% of immunized individuals (Houssiau et al., 2020). No anti–IFN-beta Abs were found. Of further interest, IFN-kinoid revealed that IFN-α blockade had an inhibitory effect on the expression of B cell associated transcripts which highlight the intricate relationship between IFN-I and the adaptive immune response (Ducreux et al., 2016).

### Anti-type I Interferon Receptor Antibodies

By targeting the common type I IFN alpha receptor 1 (IFNAR1) chain used by all IFN-I (13 IFN-alpha, IFN-beta, -delta, -epsilon, -kappa, and -omega), it is expected to obtain a broader IFN-I inhibition than anti–IFN-alpha mAbs and IFN-kinoid. Anifrolumab (MEDI546) is a fully human IgG1κ, effector null, monoclonal antibody directed against IFNAR1. *In vitro*, anifrolumab was shown to block IFN-I–dependent STAT1 phosphorylation and IFN-dependent signaling induced by IFNs and serum of patients with SLE. Anifrolumab suppressed IFN-I production by blocking the IFN autoamplification loop and inhibited pro-inflammatory cytokine induction and the upregulation of costimulatory molecules on stimulated pDCs. Blockade of IFNAR1 suppressed plasma cell differentiation in pDC/B cell co-cultures. (Riggs et al., 2018).

*Systemic lupus erythematosus (SLE)*. In a phase 2b study (MUSE), the proportion of SLE patients who reached the SRI-4 primary outcome at week 24 were higher in those treated with anifrolumab (34.3% for 300 mg dose and 28.8% for 1,000 mg dose) than placebo (17.6%) (*p* = 0.014 and *p* = 0.063, respectively). The response was driven by the effect of anifrolumab in IFN high patients (Furie et al., 2017). Two phase 3 trials, TULIP 1 (Furie et al., 2019b) and TULIP 2 (Morand et al., 2020), testing the efficacy of anifrolumab added to the standard of care in active SLE were recently published. These trials had globally a similar design and most patients received intravenous anifrolumab (300 mg) or placebo every 4 weeks for 48 weeks. In TULIP 1, the proportion of patient reaching the SRI-4 primary outcome were similar between anifrolumab 300 mg (65 [36%] of 180) and placebo (74 [40%] of 184; difference −4·2 [95% CI: −14·2 to 5·8], *p* = 0·41). However, a BICLA response was achieved by 37% patients receiving anifrolumab vs. 27% receiving placebo (difference 10·1% [95% CI 0 6–19·7]). Conversely, in TULIP 2, BICLA response used as primary outcome was reached by 47.8% in the anifrolumab group and 31.5% in the placebo group (difference, 16.3% [95% CI: 6.3–26.3]; *p* = 0.001). In TULIP 2, the proportion of patients reaching the SRI-4 was also significantly higher in the anifrolumab group with a difference than placebo of 18.2% [95% CI: 8.1, 28.3] (Morand et al., 2020). In TULIP 1, the response at week 52 in patients within IFN gene signature high did not differ between the anifrolumab and placebo groups. In TULIP 2, 48.0% in the anifrolumab group and 30.7% in the placebo group, were responders in patients with a high IFN gene signature. The respective figures in patients with a low IFN gene signature were 46.7 and 35.5%, respectively. Of note, the frequency of IFN signature high patients in the aforementioned trials was >75%, conferring higher statistical power to the analysis of these patient groups. Across all clinical trials targeting IFN-I in SLE, significant efficacy was particularly evident on lupus mucocutaneous manifestations. Across all clinical trials, *herpes zoster* and respiratory tract infections were significantly higher or tended to be higher in patients receiving active compounds than patients receiving placebo.

*Systemic sclerosis (SSc).* A phase 1 open-label trial was conducted with anifrolumab in 34 adult SSc patients. In this study, anifrolumab rapidly induced a reduction of IFN gene signature in IFN-high individuals both in blood and skin (Goldberg et al., 2014). SSc patients with high IFN-I signature had significantly higher skin thickness than IFN-low patients, suggesting an association between high IFN-I signature and SSc severity. Moreover, anifrolumab administration was associated with significant downregulation of T cell-associated proteins and upregulation of type III collagen degradation marker, but no data on clinical findings were reported (Guo et al., 2015). To our knowledge, no other treatment targeting directly IFN-I is under development in SSc.

### Targeting IFN-Beta

A small phase 2 randomized placebo-controlled trial evaluating the effect of PF-06823859 an IFN-beta 1 blocker is ongoing in dermatomyositis (clinical trial NCT03181893). The rationale of this unique study may rely on findings, indicating that serum IFN-beta rather than IFN-alpha levels appear to correlate with cutaneous manifestations and their severity in dermatomyositis (Huard et al., 2017).

### Targeting pDCs

The cells with the highest potential for IFN-alpha production are pDCs, and they play a central role in SAD pathogenesis. Therapeutic interventions aiming at decreasing pDC numbers or functions may result in decreased IFN-alpha production, in addition to decreased production of many inflammatory cytokines and decreased availability of co-stimulatory molecules. BIIB059 is a mAb that binds to BDCA2, an inhibitory receptor expressed on pDC surface, and induces its rapid internalization inhibiting the production of IFN-I. In a phase 1 trial including 12 SLEs, BIIB059 decreased the expression of IFN response genes in blood and improved the Cutaneous Lupus Erythematosus Disease Area and Severity Index Activity (CLASI-A) (Furie et al., 2019a). LILAC (NCT02847598) was a 2-part, phase 2 study investigating the efficacy and safety of BIIB059 was presented at the ACR Convergence 2020 meeting. BIIB059 (50, 150, and 450 mg) or placebo was subcutaneously administered once every 4 weeks in patients with cutaneous lupus erythematosus (CLE) with or without SLE, and all doses showed higher percent changes in CLASI-A than placebo (mean difference ranging from −24.29 to −33 (Werth et al., 2020b). A phase 3 study of BIIB059 is expected to start soon in cutaneous lupus erythematosus (CLE) with or without associated SLE.

### Targeting Signaling Initiated by TLRs and IFN-I Receptor Occupancy

Molecules targeting signaling initiated by TLRs and IFN-I receptor occupancy which have reached at least phase 2 in clinical development are reported in Table 4.

**TABLE 4:** Current phase of development of Janus kinase inhibitors in systemic autoimmune diseases (based on https://clinicaltrials.gov/accessed on November 11, 2020).

Molecule name; [main target(s) of inhibition]	SLE	SS	DM/PM	SSc	RA
Baricitinib [JAK1, 2]	Phase 3	——	Phase 2 NYR	—	Approved
	R				
Filgotinib [JAK1]	Phase 2	Phase 2	—	—	Approved
	R	R			
Peficitinib [JAK1, 2, 3]	—	—	—	—	Approved
Tofacitinib [JAK 1, 2, 3]	—	—	Phase 1	Phase 1/2	Approved
			C	C	
Upadacitinib [JAK1, (2)]	Phase 2	—	—	—	Approved
	R				
Ruxolitinib [JAK1, 2]	—	—	—	—	—

C: completed; DM: dermatomyositis; JAK: Janus kinase; NYR: not yet recruiting; PM: polymyositis, R: recruiting; RA: rheumatoid arthritis; SS: Sjogren syndrome; SSc: systemic sclerosis.

### JAK Inhibitors

Currently several JAK inhibitors (JAKis) based on small molecules are being developed in SADs including tofacitinib, baricitinib, upadacitinib, filgotinib, itacitinib, and peficitinib. Tofacitinib has been approved for RA, PsA, and ulcerative colitis, and other JAKis have been approved for RA. JAKis are also under development in phase 3 studies for atopic dermatitis, psoriasis, and alopecia areata (Nash et al., 2020). The current stages of development of main JAKi under development in SADs are presented in Table 4.

*Systemic lupus erythematosus (SLE)*. In a SLE phase 2 randomized controlled trial, baricitinib treatment induced significant reduction in the RNA expression of a network of genes associated with the JAK/STAT pathway, cytokine signaling, and SLE pathogenesis. In addition, baricitinib consistently reduced serum levels of two key cytokines implicated in SLE pathogenesis, IL-12p40, and IL-6 (Dörner et al., 2020). Baricitinib at 4 mg/day but not at 2 mg/day showed higher resolution of SLEDAI arthritis and rash at week 24 than placebo: 70 (67%) of 104 patients receiving baricitinib 4 mg vs. 56 (53%) in the placebo group (odds ratio [OR] vs. placebo 1·8, 95% CI: 1.0–3.3; *p* = 0·0414). Of note, serious infections were reported in six (6%) patients with baricitinib 4 mg, two (2%) with baricitinib 2 mg, and one (1%) with placebo (Wallace et al., 2018). Three phase 3 trials with baricitinib (NCT03843125, NCT03616912, and NCT03616964) are ongoing in SLE patients. Other JAKis are in early development phase or failed to demonstrate efficacy in SLE. Indeed, tanzisertib (JAK1/JAK2/JAK3 inhibitors) and solcitinib (JAK1 selective inhibitor) development were stopped in phase 2 (Jiang et al., 2020). Tofacitinib (a pan JAKi) with high degree of selectivity against JAK1 and JAK3 more than TYK2 or JAK2 is in early phase 2 development in CLE (NCT03288324 and NCT03159936, recruiting). Filgotinib used in association with lanraplenib (Syk inhibitor) in a phase 2 randomized controlled trial failed to demonstrate superiority over placebo (unpublished data). Finally, upadacitinib (JAK1, +/−JAK2) inhibitor is currently in a phase 2 study (recruiting) with or without association with elsubrutinib (BTK inhibitor).

*Sjögren syndrome (SS).* In the systematic review of ongoing clinical trial in SS, Felten et al. identified that JAKi may be a potential treatment in SS. Indeed, filgotinib a selective JAK1 inhibitor (Mease et al., 2018) is being evaluated in an ongoing phase 2 trial. In the same trial, two other arms are evaluating GS-9876, a (Syk inhibitor) and tirabrutinib (BTKi).

*Rheumatoid arthritis (RA).* As introduced earlier, tofacitinib, baricitinib, upadacitinib, filgotinib, and peficitinib showed efficacy in phase 3 clinical trials in RA (Jiang et al., 2020). However, the main mechanism of action of JAKi in RA is probably not related to IFN-I inhibition. Syk inhibitors [lanraplenib (GS-9876)] and BKT inhibitors [spebrutinib (CC-292) and ABBV-105] are currently in phase 2 of development (Jiang et al., 2020).

*Myositis.* It has been shown that the IFN-I pathway is dysregulated in dermatomyositis inducing decreased myotube differentiation and endothelial dysfunction (Ladislau et al., 2018). *In vitro* study and preclinical data in four DM patients showed that ruxolitinib (a JAK1/JAK2 inhibitor) decreased IFN-I scores and improved skin manifestations (Ladislau et al., 2018). A retrospective case-control series suggested that tofacitinib may be a promising treatment in rapidly progressive interstitial lung disease in anti-MDA5 DM patients and may reduce mortality (Chen et al., 2019). A long-term extension study of a 12-week open-label trial of 10 subjects with refractory dermatomyositis treated with tofacitinib (NCT03002649) has been presented at the ACR 2020 Convergence meeting showing potential promising results. For example, the mean baseline Cutaneous Dermatomyositis Activity and Severity Index (CDASI) was 25.4 ± 15 which dropped to 3 5.43 ± 2.51 by week 68 (*p* = 0.01) (Paik et al., 2018; Paik et al., 2020).

*Systemic sclerosis (SSc).* A phase 1/2 placebo-controlled trial of tofacitinib in 15 patients with SSc was reported in the ACR 2019 meeting showing that at 6 months, skin involvement and the Combined Response Index in Systemic Sclerosis (CRISS) tended to better improvement in the tofacitinib group than controls (Khanna et al., 2019).

### Other Treatments

Other treatments targeting TLRs or IFN-I downstream pathways included Syk inhibitors, Tyk2 inhibitors, TLR7, nine inhibitors, and IRF inhibitors (Chasset and Arnaud, 2018; Goess et al., 2019; Haselmayer et al., 2019; Blomgren et al., 2020; Jiang et al., 2020; Lorenzo-Vizcaya et al., 2020; Song et al., 2020), but most of these molecules are at most in early phase 2 development.

### Lessons From Therapeutic Trials Targeting IFN-I in SADs

The immunopathogenic events leading to full blown SADs are obviously complex and imprecisely delineated. Nonetheless, as reviewed here by us and elsewhere by others (Hall and Rosen, 2010; Ivashkiv and Donlin, 2014; Muskardin and Niewold, 2018; Crow et al., 2019; Rönnblom and Leonard, 2019) a very large body of evidence points to a central role of IFN-I in the pathogenesis of SADs, in particular of SLE. Could the results obtained in controlled and large clinical trials provide additional information on the role of IFN-I in SADs, maybe better to say in SLE? The issue is complicated by the difficulty of capturing clinical responses in systemic disorders where multiple parameters have to be taken into account including pathogenic mechanisms, type and extent of organ involvement, concomitant use of other drugs, disease duration, and accumulated damage at time of evaluation (Thanou et al., 2014; de Jong et al., 2016; Touma and Gladman, 2017; Merrill et al., 2018). Further, it is very likely that in SLE, various pathogenic mechanisms may contribute differentially to different disease manifestations in different individuals (Banchereau et al., 2016).

The largest body of evidence currently available to address the role of IFN-I in SADs inferred by the use of inhibitors in humans has been generated in trials assessing the efficacy of anifrolumab evaluated in three trials: the MUSE phase 2b study and the phase 3 studies TULIP 1 and TULIP 2 (Furie et al., 2017; Furie et al., 2019b; Morand et al., 2020). They provided contrasting results with one of the studies (TULIP 1) not reaching its primary endpoint. One of the main differences between these trials was the choice of different primary endpoint measures (SRI-4 based on SLEDAI for MUSE and TULIP 1 and BICLA based on BILAG for TULIP 2). For the main characteristics of activity and response indexes used in SLE, *see* Table 4. Discrepancies observed could be explained at least in part by the fact that various elements of SLE activity are weighted differently between SLEDAI and BILAG and differentially affected by anifrolumab. In addition, BILAG index captures partial responses, whereas SLEDAI captures only complete responses, and SLEDAI but not BILAG incorporate biological parameters. Overall, taking into account five of the six primary and key secondary end points, the results favored anifrolumab over placebo suggesting clinical efficacy (Salmon and Niewold, 2019). However, the effect size remained quite low, a result which is difficult to reconcile with the potential role of IFN-I in the afferent phase of the immune response in SLE. In this respect, it is interesting to note that anifrolumab seemed to be particularly efficacious on cutaneous manifestation of SLE, and an improvement of 50% of the CLASI activity used as secondary outcome was positive in TULIP 2 and showed trends in TULIP 1 (*p* = 0.054) (Furie et al., 2019b; Morand et al., 2020). Moreover, a *post hoc* analysis of TULIP 1 and 2 showed that among patients with CLASI activity ≥10 (moderate to severe skin activity) (Klein et al., 2011), CLASI-A response (≥50% reduction) was achieved by week 12 in 46.0% (49/107) of patients receiving anifrolumab vs. 24.9% (24/94) receiving placebo (difference 21.0; 95% CI 8.1%, 34.0%; nominal *p* < 0.001). Moreover, time to CLASI-A response sustained to week 52 favored anifrolumab in TULIP 1 [hazard ratio (HR) 1.91; 95% CI 1.14, 3.27] and TULIP 2 (HR 1.55; 95% CI 0.87, 2.85) (Werth et al., 2020a). Thus, evidence points to a role for IFN-I in the efferent phase of the immune response, in particular in mediating skin disease in SLE. Consistently with this conclusion, alternative treatment strategies targeting IFN-I in the most advanced phase of development including baricitinib (JAKi, active phase 3) and BIIB059 (anti-BDCA2 mAB, ongoing phase 3) have shown efficacy on skin manifestations of SLE (Wallace et al., 2018; Furie et al., 2019a).

## Conclusion

All IFNs are intimately involved in the pathogenesis of systemic autoimmunity. Most attention has been given to IFN-I. IFN-I self-stimulatory and amplificatory activity leading to high levels of ISG in SADs may indeed profoundly affect immunopathology participating both to the immune response and to tissue damage (summarized in Supplementary Figure S2). Polymorphisms in gene coding for factors participating to intracellular signaling leading to IFN production or initiated by IFNs are associated with an increased risk of disease development across the diverse SADs and disease severity. Evidence however supports models in which the production of IFN-I may be preceded by other events including the production of autoAb and IFN-gamma consistently with a preceding adaptive immune response. It is also possible that PMN, or a subset of PMN, could drive or enhance the production of IFN-I. The three-partite participation of IFN-I, autoAb, and PMN cells may explain, at least in part, the chronic nature of SADs and their wax and waning course, particularly in SLE. From the therapeutic point of view, the blockade of IFN-I biological activity aiming at reducing immunopathology in SADs makes obvious sense. The inconstant results obtained up to now applying this therapeutic strategy to SLE and other SADs highlight the complexities portending autoimmunity and heterogeneity in clinical manifestations. In the era in which precision medicine is becoming a reality when addressing therapeutic approaches in oncology, we realize that understanding autoimmunity requires further investigation to subset patients with SADs in order to offer them personalized and efficacious therapies. The IFN gene signature represents an interesting biomarker to select individuals with SADs for targeted therapeutic approaches.

## Abbreviations

IFN, interferon; IFNAR, interferon-alpha/beta receptor; IFNGR, interferon-gamma receptor; IFNLR, interferon lambda receptor; IRF, interferon regulatory factor; JAK, Janus kinase; STAT, signal transducer and activator of transcription; Tyk, tyrosine kinase. Modified from (Hall and Rosen, 2010).
