# Supplementary material for: Type I Interferons in Systemic Autoimmune Diseases: Distinguishing Between Afferent and Efferent Functions for Precision Medicine and Individualized Treatment
Source: Front Pharmacol. 2021 Apr 14;12:633821. doi: 10.3389/fphar.2021.633821 (PMC8112244; doi:10.3389/fphar.2021.633821)
Supplement: Supplementary file 1 [file Image1.tiff]

Frontiers | Type I interferons in systemic autoimmune diseases: distinguishing between afferent and efferent functions for precision medicine and individualized treatment | Pharmacology


- About
- Journals
- Research Topics
- Articles
- More

Submit

My Frontiers

Office

- TSOF
  - TSOF
  - Article Production

Typesetter 3

frontiersproduction@tnq.co.in

- Profile
- Settings & Privacy
- Help Center
- Logout

Submit

**Impact Factor 4.225** | **CiteScore 5.0**More on impact ›

|  |  |
| --- | --- |
| Frontiers in Pharmacology | Inflammation Pharmacology |

Toggle navigation


Section


- (current)Section
- About
- Articles
- Research topics
- For authors 
  - Why submit?
  - Fees
  - Article types
  - Author guidelines
  - Review guidelines
  - Submission checklist
  - Contact editorial office
  - Submit your manuscript
- Editorial board

- *Article alerts*

##### This article is part of the Research Topic

Precision Medicine and Immuno-Mediated Inflammatory Diseases:
Latest Progress and Next Challenges
View all
5
Articles

Articles


**Suggest a Research Topic >**

- 61
  total views

 View Article Impact

**Suggest a Research Topic >**

##### SHARE ON

- Facebook

  0
- Twitter

  0
- LinkedIn

  0
- AddThis

  New


## Review ARTICLE

Front. Pharmacol.
| doi: 10.3389/fphar.2021.633821

# Type I interferons in systemic autoimmune diseases: distinguishing between afferent and efferent functions for precision medicine and individualized treatment Provisionally accepted The final, formatted version of the article will be published soon. **Notify me**

Carlo Chizzolini1\*,  François Chasset2 and  Jean-Michel Dayer3

- 1Department of Pathology and Immunology, Faculty of Medicine, University of Geneva, Switzerland
- 2Sorbonne Universités, France
- 3Université de Genève, Switzerland

A sustained increase in type-I interferons (IFN-I) may accompany clinical manifestations and disease activity in systemic autoimmune disorders (SADs). Despite the very frequent presence of IFN-I in SADs, clinical manifestations are extremely varied between and within SADs. The present short review will address the following key questions associated with high IFN-I in SADs in the perspective of precision medicine. 1. What are the mechanisms leading to high IFN-I? 2. What are the predisposing conditions favoring high IFN-I production? 3. What is the role of IFN-I in the development of distinct clinical manifestations within SADs? 4. Would therapeutic strategies targeting IFN-I be helpful in controlling or even preventing SADs? In answering these questions, we will underlie areas of incertitude and the intertwined role of autoantibodies, immune complexes, and neutrophils.

Keywords: 
interferon, systemic lupus erythematosus (SLE), Genetic polymorphism, interferon-stimulated genes (ISGs), Polymorphonuclear neutrophils (PMN), Keratinocytes, autoantibody (autoAb), Systemic autoimmune diseases (SADs)

Received: 26 Nov 2020;
Accepted: 03 Mar 2021.

Copyright: © 2021 Chizzolini, Chasset and Dayer. This is an open-access article distributed under the terms of the Creative Commons Attribution License (CC BY). The use, distribution or reproduction in other forums is permitted, provided the original author(s) and the copyright owner(s) are credited and that the original publication in this journal is cited, in accordance with accepted academic practice. No use, distribution or reproduction is permitted which does not comply with these terms.

\* Correspondence: 
Prof. Carlo Chizzolini, Department of Pathology and Immunology, Faculty of Medicine, University of Geneva, Geneva, CH - 1211, Geneva, Switzerland, carlo.chizzolini@unige.ch

Write a comment...

Add

##### COMMENTARY

##### ORIGINAL ARTICLE

##### People also looked at

## Liangxue Jiedu Formula Improves Psoriasis and Dyslipidemia Comorbidity via PI3K/Akt/mTOR Pathway

Xinran Xie, Lei Zhang, Xue Li, Weihong Liu, Ping Wang, Yan Lin, Xuyang Han and Ping Li

## Deciphering the Pharmacological Mechanisms of Guizhi-Fuling Capsule on Primary Dysmenorrhea Through Network Pharmacology

Siqin Zhang, Xinxing Lai, Xin Wang, Gang Liu, Zhenzhong Wang, Liang Cao, Xinzhuang Zhang, Wei Xiao and Shao Li

## Bibliometric analysis of global research trends on ultrasound microbubble: a quickly developing field

Haiyang Wu, Linjian Tong, Yulin Wang, Hua Yan and Zhiming Sun

## Systems Pharmacology Study of the Anti-Liver Injury Mechanism of Citri Reticulatae Pericarpium

Jianxiong Wu, Xietao Ye, Songhong Yang, Huan Yu, Lingyun Zhong and Qianfeng Gong

## Therapeutic Potential of Carica papaya Leaves for the Treatment of Dengue Fever: A Review Based on Scientific Evidences

Md Moklesur Rahman Sarker, Farzana Khan and Isa Naina Mohamed

**Suggest a Research Topic >**

×

#### Supplementary Material

  

There is no supplementary material currently available for this article

Loading supplemental data...

  

|  | File Name |  |
| --- | --- | --- |
|  | Table 1.pdf |  |
|  | Image 1.TIFF |  |
|  | Image 2.TIFF |  |

  

Close

- About Frontiers
- Institutional Membership
- Books
- News
- Frontiers' social media
- Contact
- Careers
- Submit
- Newsletter
- Help Center
- Terms & Conditions
- Privacy Policy

© 2007 - 2021 Frontiers Media S.A. All Rights Reserved

### Privacy Preference Center

Our website uses cookies that are necessary for its operation. Additional cookies are only used with your consent. These cookies are used to store and access information such as the characteristics of your device as well as certain personal data (IP address, navigation usage, geolocation data) and we process them to analyse the traffic on our website in order to provide you a better user experience, evaluate the efficiency of our communications and to personalise content to your interests. Some cookies are placed by third-party companies with which we work to deliver relevant ads on social media and the internet. Click on the different categories' headings to change your cookie preferences. Click on "More Information" if you wish to learn more about how data is collected and shared.
More information

### Manage Consent Preferences

#### Strictly Necessary Cookies

Always Active

These cookies are necessary for the website to function and cannot be switched off in our systems. They are usually only set in response to actions made by you which amount to a request for services, such as setting your privacy preferences, logging in or filling in forms. You can set your browser to block or alert you about these cookies, but some parts of the site will not then work. These cookies do not store any personally identifiable information.

#### Analytics Cookies

Analytics Cookies

These cookies allow us to count visits and traffic sources so we can measure and improve the performance of our site. They help us analyse which pages are the most and least popular and see how visitors move around the site.    All information these cookies collect is aggregated and therefore anonymous.

#### Functional Cookies

Functional Cookies

These cookies enable the website to provide enhanced functionality and personalisation. They may be set by us or by third party providers whose services we have added to our pages. If you do not allow these cookies then some or all of these services may not function properly.

#### Advertising Cookies

Advertising Cookies

These cookies may be set through our site by our advertising partners. They may be used by those companies to build a profile of your interests and show you relevant adverts on other sites.    They do not store directly personal information, but are based on uniquely identifying your browser and internet device. If you do not allow these cookies, you will experience less targeted advertising.

### Back Button Performance Cookies

Vendor Search  Search Icon

Filter Icon

Clear

checkbox label label

Apply Cancel

Consent Leg.Interest

checkbox label label

checkbox label label

checkbox label label

Confirm My Choices
