# Supplementary material for: Type I Interferons in Systemic Autoimmune Diseases: Distinguishing Between Afferent and Efferent Functions for Precision Medicine and Individualized Treatment
Source: Front Pharmacol. 2021 Apr 14;12:633821. doi: 10.3389/fphar.2021.633821 (PMC8112244; doi:10.3389/fphar.2021.633821)
Supplement: Supplementary file 3 [file table1.pdf]

**Supplementary Table S1. Indexes used to define disease activity and response to treatment in SLE**

| | | | |
|---|---|---|---|
| SLEDAI[1] | SLE disease activity index | Based on the sum of values attributed to 24 differentially weighted descriptors of clinical and laboratory items | (Bombardier et al., 1992) |
| BILAG[1] | British Isles Lupus Assessment Group. | Grade form **A** very active, **B** active, **C** moderately active, **D** was active, **E** never active 9 domains: constitutional, mucocutaneous, central nervous system, musculoskeletal, cardiovascular/respiratory, abdominal, renal, ophthalmic and hematological | (Isenberg et al., 2005) |
| CLASI | Cutaneous Lupus Erythematosus Disease Area and Severity Index | Measurement instrument for mucocutaneous lupus erythematosus lesions with separate scores for damage and activity. | (Albrecht et al., 2005) |
| SRI[2] | SLE responder index | Composite index that utilizes SLEDAI score to determine global improvement; BILAG domain scores to ensure no significant worsening; and Physician's Global Assessment (PGA) to ensure that improvements in disease activity are not achieved at the expense of the patient health | (Furie et al., 2009) |
| BICLA[3] | BILAG-based Composite Lupus Assessment | Composite index that utilizes BILAG-index to determine improvement, SLEDAI score to ensure no significant worsening, and PGA to ensure that improvements in disease activity are not achieved at the expense of the patient health | (Wallace et al., 2011), (Wallace et al., 2014) |

1. SLEDAI and BILAG exist in several versions with differences in subtle characteristics, which go beyond the scope of this review.
2. In SRI the improvement in SLEDAI can be gauged at 4, 6, etc points and referred as: SRI (4), SRI (6)
3. Requires improvement in all A and B scores.


Albrecht, J., Taylor, L., Berlin, J.A., Dulay, S., Ang, G., Fakharzadeh, S., Kantor, J., Kim, E., Militello, G., Mcginnis, K., Richardson, S., Treat, J., Vittorio, C., Van Voorhees, A., and Werth, V.P. (2005). The CLASI (Cutaneous Lupus Erythematosus Disease Area and Severity Index): an outcome instrument for cutaneous lupus erythematosus. *J Invest Dermatol* 125**,** 889-894.

Bombardier, C., Gladman, D.D., Urowitz, M.B., Caron, D., and Chang, C.H. (1992). Derivation of the SLEDAI. A disease activity index for lupus patients. The Committee on Prognosis Studies in SLE. *Arthritis Rheum* 35**,** 630-640.

Furie, R.A., Petri, M.A., Wallace, D.J., Ginzler, E.M., Merrill, J.T., Stohl, W., Chatham, W.W., Strand, V., Weinstein, A., Chevrier, M.R., Zhong, Z.J., and Freimuth, W.W. (2009). Novel evidence-based systemic lupus erythematosus responder index. *Arthritis Rheum* 61**,** 1143-1151.

Isenberg, D.A., Rahman, A., Allen, E., Farewell, V., Akil, M., Bruce, I.N., D'cruz, D., Griffiths, B., Khamashta, M., Maddison, P., Mchugh, N., Snaith, M., Teh, L.S., Yee, C.S., Zoma, A., and Gordon, C. (2005). BILAG 2004. Development and initial validation of an updated version of the British Isles Lupus Assessment Group's disease activity index for patients with systemic lupus erythematosus. *Rheumatology (Oxford)* 44**,** 902-906.

Wallace, D.J., Kalunian, K., Petri, M.A., Strand, V., Houssiau, F.A., Pike, M., Kilgallen, B., Bongardt, S., Barry, A., Kelley, L., and Gordon, C. (2014). Efficacy and safety of epratuzumab in patients with moderate/severe active systemic lupus erythematosus: results from EMBLEM, a phase IIb, randomised, double-blind, placebo-controlled, multicentre study. *Ann Rheum Dis* 73**,** 183-190.

Wallace, D.J., Strand, V., Furie, R., Petri, M., Kalunian, K., Pike, M., Kelley, L., and Gordon, C. (2011). "Evaluation of treatment success in systemic lupus erythematosus clinical trials: development of the British Isles Lupus Assessment Group-based composite lupus assessment endpoint.", in: *2011.* Arthritis Rheum).
